# Social concordance and patient reported experiences in countries with different gender equality: a multinational survey

**DOI:** 10.1186/s12875-024-02339-y

**Published:** 2024-03-23

**Authors:** D. Eggermont, A. E. Kunst, P. P. Groenewegen, R. A. Verheij

**Affiliations:** 1 School of Social and Behavioral Sciences, Tranzo Tilburg University, Heidelberglaan 1, Utrecht, 3584 CS the Netherlands; 2 Department of Public Health, Amsterdam UMC, University of Amsterdam, Meibergdreef 9, Amsterdam, 1105 AZ the Netherlands; 3 Nivel, Netherlands Institute for Health Services Research, Otterstraat 118-124, Utrecht, 3513 CR The Netherlands; 4 National Health Care Institute, Willem Dudokhof 1, 1112 ZA Diemen, the Netherlands

**Keywords:** Age, Gender, Concordance, Equality, Patient, Reported, Experience, Measures, Evaluation

## Abstract

**Background:**

Patient reported experiences (PREMs) are important indices of quality of care. Similarities in demography between patient and doctor, known as social concordance, can facilitate patient-doctor interaction and may be associated with more positive patient experiences. The aim of this research is to study associations between gender concordance, age concordance and PREMs (doctor-patient communication, involvement in decision making, comprehensiveness of care and satisfaction) and to investigate whether these associations are dependent on a countries’ Gender Equality Index (GEI).

**Methods:**

Secondary analysis on a multinational survey (62.478 patients, 7.438 GPs from 34 mostly European countries) containing information on general practices and the patient experiences regarding their consultation. Multi-level analysis is used to calculate associations of both gender and age concordance with four PREMs.

**Results:**

The female/female dyad was associated with better experienced doctor-patient communication and patient involvement in decision making but not with patient satisfaction and experienced comprehensiveness of care. The male/male dyad was not associated with more positive patient experiences. Age concordance was associated with more involvement in decision making, more experienced comprehensiveness, less satisfaction but not with communication. No association was found between a country’s level of GEI and the effect of gender concordance.

**Conclusion:**

Consultations in which both patient and GP are female are associated with higher ratings of communication and involvement in decision making, irrespective of the GEI of the countries concerned. Age concordance was associated with all PREMs except communication. Although effect sizes are small, social concordance could create a suggestion of shared identity, diminish professional uncertainty and changes communication patterns, thereby enhancing health care outcomes.

**Supplementary Information:**

The online version contains supplementary material available at 10.1186/s12875-024-02339-y.

## Introduction

Patient reported experience measures (PREMs) are health care quality indices derived from the perspective of patients. Although patient experiences are distinct from more classic measures for clinical effectiveness (e.g. decrease in blood pressure) and patient safety, these measures are interrelated [1, 2]. Therefore, understanding what improves or undermines patient experiences can help achieve treatment goals and improve quality of care.

Social concordance is a concept of similarity based on demographic characteristics. Most research in this field has focused on the influence of ethnic concordance, gender concordance or language concordance on health care consultation outcomes. Studies focusing on gender concordance are inconclusive whether PREMs are affected by the doctor-patient gender composition. For example, female gender concordance can be associated with better communication [3], higher health care provider score [4], more harmonious connection [5] and more agreement on advice [6]. Gender concordance is also associated with patients experiencing more overall satisfaction [7], although this finding is refuted in a large comprehensive American study [8]. This study, containing over 100.000 patient experience survey’s, showed no association between gender concordance and Press Ganey scores (reflecting the likelihood of recommending the physician to others). Also, other studies find that gender concordance is not related to experienced participatory decision-making, encounter quality, trust, quality of life or quality of care [9–13]. Research on age concordance is scarce, but existing evidence suggests associations with enhanced health care quality perceptions [7]. In conclusion, whether doctor-patient concordance regarding gender and age can influence PREMs is still under debate.

In this study, we will investigate the influence of both gender concordance and age concordance on doctor-patient communication, patient involvement in decision making, experienced comprehensiveness of care and patient satisfaction in primary care. It is hypothesized that gender and age concordance are associated with higher scores on PREMs.

The extent to which gender and age concordance affect PREMs could also be culturally determined. In particular gender, which is a social construct, can have different interpretations among different countries [14]. Countries differ in gender norms (e.g. masculinity norms or ideas how man and women should relate to each other) [15], possibly influencing how gender concordance affects patient-doctor interaction. Therefore, we will investigate whether associations between gender concordance and PREMs vary between countries with a different degree of gender equality. It is hypothesized that in countries with a lower gender equality index (GEI), gender stereotypes are stronger resulting into a higher likelihood of gender concordance/discordance influencing PREMs. This aligns with the convergence hypothesis, which states that health inequalities between man and women decrease as gender equality increases [16, 17].

## Methods

### Data source

For this study, the ‘Quality and Costs of Primary Care in Europe’ database (QUALICOPC) is used, provided by the Netherlands Institute for Health Services Research (Nivel). The QUALICOPC is a multinational survey held among GPs and their patients, aimed at gathering extensive information on the participating general practices, the professional behavior of GPs and the experiences of their patients [18, 19]. Recently, this database has also been used by other researchers studying the association of migration concordance with PREMs [20].

### Data collection procedure

Between 2011 and 2013, data was collected across 31 European countries, Australia, Canada and New Zealand. The primary objective was to establish a nationally representative sample of GPs in each participating country, with a predefined target sample size of 220 GPs per country (Cyprus, Iceland, Luxembourg and Malta had a target of 80 GPs). In most countries (19/34), this was achieved by drawing a random list sample from the national register of GPs. To prevent clustering, only one GP per practice was included. In countries where a national register was not available (5/34), a multistage sampling procedure was used. This involved combining registers from different regions within the country and subsequently selecting GPs through a random process. Notably, in larger countries with discernible variations in healthcare systems across regions (4/34), GPs were exclusively sampled from nationally representative regions. Conversely, in smaller countries (3/34), the entire GP population was approached for participation. In Italy and Norway, GPs were included by opportunity sampling. The overall mean response rate was 38%. Importantly, the participating GPs demonstrated representativeness concerning age and gender in comparison to the broader GP population within their respective countries [21].

In each participating practice, one GP and nine adult patients filled in a questionnaire. Patients who just had a face to face consultation with their GP were consecutively invited by trained fieldworkers to fill in a questionnaire about their experiences resulting from the consultation. This was continued until nine questionnaires per practice were collected. Participation was voluntary and informed consent was obtained. The average response rate of patients was 74%. In total, 63.887 patients completed a questionnaire and 7.438 GPs completed a questionnaire. 62.478 (97.8%) of the patient experience questionnaires were successfully matched with the GP questionnaires. All included respondents were 18 years or older and all information was made anonymous. More details about the study protocol, the questionnaire background and survey design have been published elsewhere [21–23].

### Variables

Of the questionnaire directed to the GPs, only the information concerning gender, age and practice location (city/suburbs/town/urban–rural/rural) was used. The questionnaire directed to the patients consisted of statements which could be answered yes/no and were designed to measure, amongst others, the latent variables ‘doctor-patient communication’ (5 items) and ‘comprehensiveness of care’ (2 items). Moreover, 2 items were selected which were interpreted as measuring ‘patient satisfaction’. The variable ‘patient involvement in decision making’ was measured with one item (dichotomous). For the multiple item latent variables, scales were constructed using multilevel latent variable analyses in a four-level model (items nested within patients, nested within GPs, nested within countries). The reliability of these scales are reported in Appendix A. Gender concordance was considered a four category variable, containing these dyads: male/male, male/female, female/male and female/female (gender GP/gender patient). Age concordance was defined as a maximum age difference of five years between patient and GP, which is similar to how this construct was defined in other studies [7, 24]. Age discordance was divided into two groups: patients being substantially younger than their GP (‘younger patient/older GP’ dyad) and more involvement in decision making and patients being substantially older than their GP (‘older patient/younger GP’ dyad).

Countries were given a score on the gender equality index (GEI) using the indexation of 2015 published by the European Institute of Gender Equality [25], based on datapoints originating from 2012. No GEI scores were available for Iceland, Norway, Switzerland, Turkey, Australia, Canada, New Zealand and FYR Macedonia. Scores ranged from 50.10 (Greece) to 79.70 (Sweden), with higher scores corresponding with more gender equality. Countries were categorized into ‘low GEI’ (index < 55), ‘average GEI’ (index 55–65) and ‘high GEI’ (index > 65). Table 1 shows all countries and their corresponding GEI.

**Table 1:** Categorization of countries based on Gender Equality Index (GEI)^a^

Low GEI (< 55) *n* = 13.323	Average GEI (55–65) *n* = 15.340	High GEI (> 65) *n* = 16.850
Cyprus (*n* = 603) – 50.6	Austria (*n* = 1592) – 61.3	Belgium (*n* = 3619) – 70.2
Estonia (*n* = 1120) – 53.5	Bulgaria (*n* = 1971) – 56.9	Denmark (*n* = 1855) – 75.6
Greece (*n* = 1953) – 50.1	Czech Republic (*n* = 1971) – 56.7	Finland (*n* = 1189) – 74.4
Hungary (*n* = 1925) – 51.8	England (*n* = 1279) – 58.0	Ireland (*n* = 1473) – 67.7
Lithuania (*n* = 1963) – 54.2	Germany (*n* = 2117) – 64.9	Luxembourg (*n* = 662) – 65.9
Portugal (*n* = 1877) – 54.4	Italy (*n* = 1902) – 56.5	Netherlands (*n* = 1906) – 74.0
Romania (*n* = 1966) – 51.2	Latvia (*n* = 1936) – 56.2	Slovenia (*n* = 1747) – 66.4
Slovakia (*n* = 1916) – 52.4	Malta (*n* = 626) – 57.8	Spain (*n* = 3670) – 67.4
	Poland (*n* = 1946) – 56.9	Sweden (*n* = 729) – 79.7

^a^For Australia (*n* = 1190), Canada (*n* = 6813), FYR Macedonia (*n* = 1283), Iceland (*n* = 685), New Zealand (1148), Norway (*n* = 1468), Switzerland (*n* = 1773) and Turkey (*n* = 2605) there were no GEI’s were available (total *n* = 16,965)

## Statistics

Due to clustering of observations at the level of countries and GPs, we performed multi-level analysis [26], using three different data levels: patients (1) nested within GPs (2) nested withing countries (3). We predicted doctor-patient communication, patient satisfaction and comprehensiveness of care using linear regressions and patient involvement in decision making using logistic regressions (with a fixed intercept and random effects at the country and GP level). Main predictors in our models are ‘gender concordance’ and ‘age concordance’ and the control variables are ‘GP age’, ‘patient age’, ‘chronic condition’ (yes/no), ‘origin of patient’ (native/not native), ‘education’ (primary/secondary/post-secondary), ‘self-reported level of household income’ (low/average/high) and ‘location of practice’ (big city/town/mixed rural/rural). Comparisons between low, middle and high GEI countries were made by adding interaction terms (gender concordance * GEI-categories) to the model. All analyses were performed using STATA 17.0.

## Results

### Descriptive statistics

About half of the questionnaires were filled in after the patient visited a male GP (47,8%) and most of the included patients were female (61.2%). Half of the consultations were gender concordant (54.3%) and 20.2% were age concordant. All gender dyad characteristics and age dyad characteristics are reported in Table 2. Notably, the age of the patient varies greatly between the three age dyads. Prevalence of one or more chronic conditions is also higher in the dyad with older patients and the dyad with younger patients contained more higher educated individuals.

**Table 2:** Descriptive statistics of gender dyads and age dyads

	Male GP / Male patient	Male GP / Female patient	Female GP / Female patient	Female GP / Male patient	Age concordant	Age discordant younger patients	Age discordant older patients
Sample size	12,589 (20.1%)	16,824 (26.9%)	20,855 (33.4%)	11,341 (18.2%)	12,635 (20.2%)	23,392 (37.4%)	24,562 (39.3%)
Age patient – Mean (SD)	53.4 (17.5)	50.9 (17.5)	49.8 (17.2)	51.2 (17.4)	50.6 (10.0)	35.1 (9.9)	66.6 (10.9)
Age GP – Mean (SD)	51.8 (9.7)	51.6 (9.7)	49.2 (9.4)	49.1 (9.5)	50.6 (9.6)	53.7 (8.5)	47.0 (9.5)
$\ge$ 1 chronic condition—%	50.6	49.1	49.7	51.4	51.4	29.2	69.2
Native patient- %	86.5	86.3	88.8	90.9	88.2	87.8	88.4
Education							
- Primary education	28.1	29.6	26.6	25.9	24.1	17.1	39.4
- Upper secondary	40.1	38.9	35.9	40.9	38.6	42.1	35.1
- Post-secondary	31.9	31.6	37.5	33.2	37.3	40.8	25.5
Income							
- Below average	26.4	29.7	33.2	31.3	28.6	27.4	34.3
- Average	57.8	60.1	56.0	53.6	57.1	59.4	55.0
- Above average	15.9	10.2	10.9	15.1	14.6	13.2	10.7

**Fig. 1:**
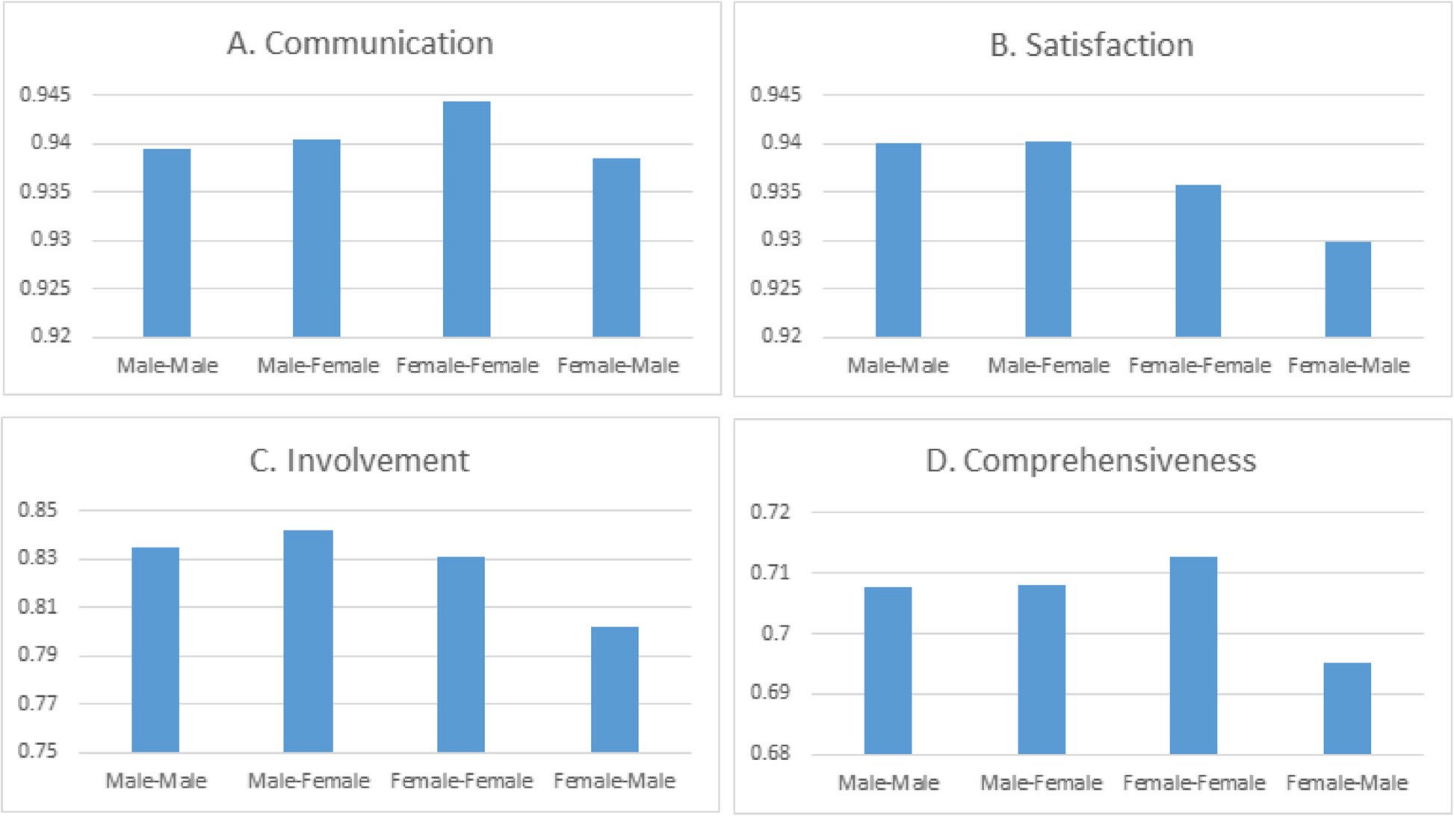
Score for each PREM per gender dyad

**Fig. 2:**
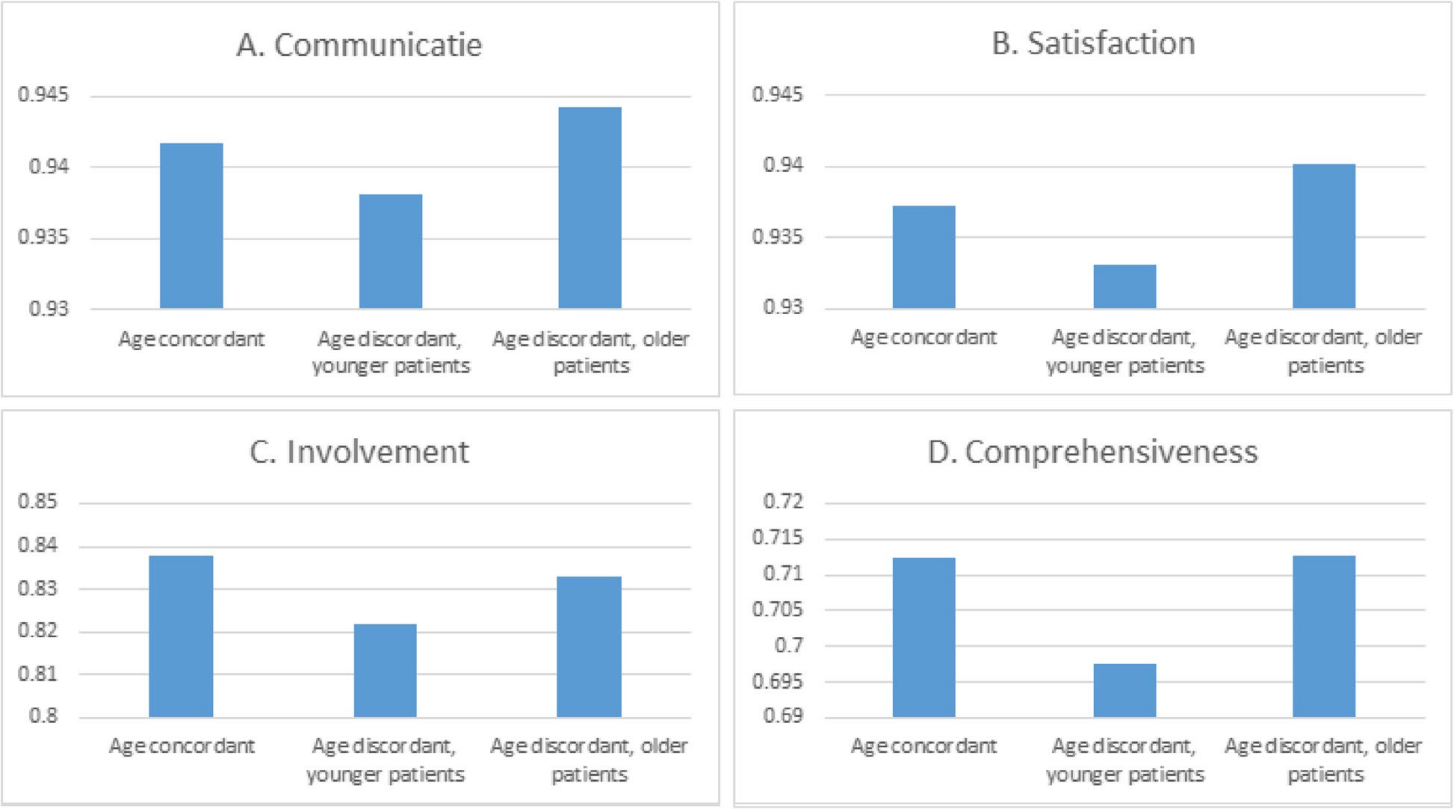
Score for each PREM by age dyad

Scores for communication, satisfaction, involvement and comprehensiveness were negatively skewed (towards high scores). Figure 1 shows that especially in consultations with a female GP, there are notable differences in PREM-scores between concordant and discordant gender dyads. In consultations with a male GP, scores given by male and female patients are more or less equal. Figure 2 shows that patients aged younger than their GP report the lowest scores on communication, satisfaction, involvement and comprehensiveness, whereas patients aged older than their GP report better communication and higher satisfaction.

### Main results

Female concordance is associated with higher scores on communication (*p* ≤ 0.01) and more involvement in decision making (*p* ≤ 0.01) compared to the other dyads (Table 3). The female/female dyad is associated with higher patient satisfaction compared to the male/female dyad (*p* = 0.01) and the male/male dyad (*p* = 0.02), but not compared to the female/male dyad. Findings are similar for comprehensiveness of care: the female/female dyad is associated with higher scores compared to both dyads with a male GP (*p* = 0.00), but not compared to the female/male dyad. Patient satisfaction and comprehensiveness of care are thus associated with the gender of the GP (higher scores when the GP is female) and not with gender concordance.

**Table 3:** Multi-level multivariate regression predicting the associations between gender and age concordance on four PREMs

(*n* = 62,478)	**Communication**	**Satisfaction**	**Involvement** ^**a**^	**Comprehensiveness**
	Coeff (SE)	Coeff (SE)	OR (SE)	Coeff (SE)
Age patient	-0.04 (0.02)	0.02 (0.02)	1.00 (0.00)	0.04 (0.02)*
Age GP	-0.16 (0.07)*	-0.09 (0.06)	1.00 (0.00)	0.11 (0.19)
Gender concordance (ref = female/female)				
•Female GP/male patient	-1.11 (0.41)*	0.15 (0.60)	0.89 (0.04)*	-0.69 (0.000)
•Male GP/female patient	-4.77 (1.44)*	-3.55 (1.29)*	0.86 (0.03)*	-10.53 (2.74)*
•Male GP/male patient	-5.18 (1.48)*	-3.62 (1.53)*	0.79 (0.04)*	-10.67 (2.76)*
Age concordance (ref = conc)				
•Discordant young GP	-0.04 (0.41)	0.62 (0.53)	0.92 (0.03)*	-0.49 (0.49)
•Discordant old GP	0.21 (0.50)	1.69 (0.43)*	0.94 (0.04)	**-0.99 (0.42)**
Chronic condition (ref = no)	-0.28 (0.28)	-1.14 (0.32)*	1.16 (0.05)*	3.59 (0.49)*
Native (ref = yes)	-3.50 (0.78)*	-4.42 (1.03)*	0.82 (0.05)*	0.31 (0.46)
Education (ref = average)				
•Low	-2.49 (0.53)*	0.40 (0.72)	0.92 (0.04)	-0.002 (0.42)
•High	1.39 (0.37)*	1.19 (0.46)*	1.08 (0.04)*	-0.68 (0.39)*
Income (ref = average)				
•low	-2.49 (0.53)*	-2.30 (0.65)*	0.93 (0.04)	0.19 (0.34)
•high	1.39 (0.37)	0.31 (0.54)	1.17 (0.05)*	0.04 (0.43)
Practice location (ref = big city)				
•Suburbs	-1.58 (2.20)	1.25 (2.32)	1.10 (0.09)	2.40 (5.16)
•Town	-2.97 (2.39)	1.98 (2.80)	1.23 (0.07)*	3.71 (4.45)
•Mixed rural	1.52 (1.37)	4.78 (1.97)*	1.25 (0.09)*	10.53 (3.25)*
•Rural	3.37 (2.24)	8.15 (2.56)*	1.35 (0.09)*	18.86 (6.29)*
**Reduction of variance** ^**b**^				
Country level	-0.3%	4.4%	3.5%	15.7%
GP level	0.7%	2.4%	-3.3%	0.0%
**ICC's empty models**				
Country level	14.4%	21.8%	10.1%	45.5%
GP level	61.2%	52.6%	18.3%	51.4%

^*^ *p* < 0.05

^a^for the dependent variable ‘involvement’ effect sizes are reported using odds ratios because a binary logistic regression was performed

^b^reductions of variance are calculated after adding the main predictors and covariates to the models

Age concordance was not associated with experienced communication. Consultations with an older patient/younger GP dyad were associated with higher satisfaction (*p* = 0.01) and less experienced comprehensiveness (*p* = 0.02) compared to consultations with age concordance. Consultations with a younger patient/older GP dyad were associated with lower involvement scores compared to consultations with age concordance (OR 0.92, *p* = 0.02).

In contrast to the former model (Table 3), there are no associations between gender concordance and all four PREMs in both low and high GEI countries (Table 4). In both low and high GEI countries, consultations with a female GP were associated with higher scores for communication and satisfaction. Male concordance was associated with lower involvement in decision making compared to female concordance in low GEI countries whereas in high GEI countries, both dyads with a female GP were associated with higher involvement in decision. There are no associations between gender dyads and comprehensiveness of care in low GEI countries, however consultations with a female GP were associated with better experienced comprehensiveness compared to consultations with a male GP in high GEI countries. Our findings do not support the hypothesis that associations between gender concordance and PREMs are stronger in countries with a low GEI.

**Table 4:** Multi-level multivariate regression predicting the associations between gender and age concordance on four PREMs, accounting for Gender Equality Index (GEI)^a,b^

(*n* = 45,513)	**Communication**	**Satisfaction**	**Involvement** ^**c**^	**Comprehensiveness**
	Coeff (SE)	Coeff (SE)	OR (SE)	Coeff (SE)
**GEI—LOW**				
Gender dyads (ref = female/female)				
•female GP/male patient	-1.00 (0.62)	0.58 (1.15)	1.01 (0.06)	-1.10 (0.89)
•male GP/female patient	-10.83 (3.24)*	-5.62 (2.46)*	0.92 (0.04)	-5.20 (6.65)
•male GP/male patient	-12.15 (3.09)*	-7.32 (2.97)*	0.77 (0.07)*	-5.91 (6.65)
**GEI—MIDDLE**				
Gender dyads (ref = female/female)				
•female GP/male patient	-2.79 (0.70)*	-1.29 (1.14)	0.76 (0.04)*	-1.75 (1.01)
•male GP/female patient	0.67 (2.40)	-4.27 (3.69)	0.83 (0.04)*	-14.92 (5.62)*
•male GP/male patient	0.02 (2.33)	-3.57 (3.98)	0.81 (0.07)*	-15.12 (5.77)*
**GEI—HIGH**				
Gender dyads (ref = female/female)				
•female GP/male patient	-0.22 (0.62)	1.22 (0.97)	0.96 (0.08)	0.58 (0.89)
•male GP/female patient	-6.63 (1.43)*	-4.95 (2.04)*	0.83 (0.05)*	-15.53 (4.73)*
•male GP/male patient	-6.72 (1.40)*	-5.29 (2.72)*	0.79 (0.09)*	-16.25 (4.43)*
**Reduction of variance** ^**d**^				
Country level	7.0%	12%	27.1%	11.1%
GP level	2.8%	4.1%	-2.8%	1.9%
**ICC's empty models**				
Country level	10.6%	15.8%	5.3%	41.2%
GP level	63.6%	56.9%	19.3%	55.7%

^*^ *p* < 0.05

^a^The control variables were used in the calculation but are not presented in the table

^b^The model was calculated three times, for each GEI category with the female/female dyad as reference group

^c^for the dependent variable ‘involvement’ effect sizes are reported using odds ratios because a binary logistic regression was performed

^d^reductions of variance are calculated after adding the main predictors, interaction terms and covariates to the models

### Model properties

The extent to which the combination of independent variables used in the model (Table 3) explain the variance of communication, satisfaction and involvement in decision making on the country and GP level is limited (ranging from 0.3% to 4.4%). The model explains 15.7% of the variance in comprehensiveness of care on the country level. After adding the GEI variable to the model (and excluding the countries of which a GEI was not available), the variance of the communication, satisfaction and involvement in decision making was explained to a bigger extend (Table 4). This was especially true on the country level (reduction of variance ranging from 7.0% to 27.1%).

## Discussion

### Summary

Female concordance is associated with better communication and more involvement in decision making. The age concordant dyad is associated with more experienced comprehensiveness and less satisfaction (compared to the older patient/younger GP dyad) and with more involvement in decision making (compared to the younger patient/older GP dyad). Communication was not associated with age concordance. The hypothesis that countries with a lower GEI would demonstrate stronger associations between gender concordance and PREMs could not be supported.

### Strengths and limitations

The combination of a large number of respondents gathered across a large number of countries and the ability to take into account the nested nature of our respondents, strengthens the reliability of our results. The response rates among GPs and patients, at 38% and 74% respectively, introduce a risk of selection bias. Although the participating GPs generally reflected the age and gender distribution of the broader GP population in their respective countries, the representativeness regarding other characteristics, such as ethnicity, remains uncertain. Additionally, it is possible that participating GPs have greater interest in research or have more time availability compared to their non-participating counterparts, potentially influencing our study outcomes. Varying GP response rates (e.g., less than 10% in Sweden, over 70% in Spain) impact result generalizability in those particular countries. Notably, one in four patients opted not to participate, with the reasons for non-participation uncollected. Possible explanations for patient non-participation may include time constraints and privacy concerns. The latter, in particular, raises the possibility that GP visits for specific sensitive issues (e.g., psychological or sexual problems) may be underrepresented in our study sample. However, the precise impact of this possible underrepresentation on the measured patient experiences is challenging to ascertain.

The surveys contained patient experiences reported directly after consultation, which strengthens their validity. Moreover, the questions used to operationalize patient experiences were derived from various validated sources. However, all of them consisted out of yes/no answers which is less nuanced than for example Likert-scales. The distribution of scores were negatively skewed meaning most respondents reported very high scores, which is a common finding in post-consultation evaluation studies [8]. Most PREMs were measured by integrating multiple items into a scale, creating latent constructs. Although this method helps to provide more valid variables, this also makes the interpretation of scores somewhat abstract.

We used the gender equality indexation of the European Institute of Gender Equality, which is among the most comprehensive indexations available. However, this report did not contain indexations for 8 out of the 34 countries included in this study. These countries were therefore excluded in our model, lowering the statistical power.

### Comparison with existing literature

Research on gender concordance and patient experiences show mixed results. It should however be taken into account that there are a lot of derivates of patient experiences, making it challenging to compare these study results. Some studies find associations between female concordance and specific PREMs [3–7], such as physician communication or experienced agreement on advice. Other studies find no significant association between gender concordance and PREMs [8–13]. Some of these findings can be explained by lack of statistical power [9] or the use of too broad outcome variables [10]. Nonetheless, the study of Takeshita et al. [8] was large and comprehensive and showed no association between gender concordance and one specific PREM: the likelihood of recommending the physician to a friend (on a 1 to 5 scale). Although this appears to contrast our findings, it should be noted that our studied PREMs are dissimilar. Moreover, as in most studies finding significant associations, especially the female-female dyad appears to be linked with higher scores on PREMs. This distinction was not specifically reported in the study of Takeshita et al.

Associations between age concordance and PREMs are poorly studied. In an observational study, age concordance was part of the construct social concordance (also entailing race, gender and education concordance). Social concordance was associated with higher satisfaction of care, but it is not clear to what extent age concordance contributed to this finding [7]. Our finding that age concordance can be associated with patient experiences is consistent with the idea that proximity in demographics may positively influence patient experiences.

This is the first study which takes the cross-national variability of gender equality into account when focusing on gender concordance. We hypothesized that more gender equality would diminish effects of gender concordance. However, we did not find a pattern to substantiate this. Possibly, the impact of specific gender dyads on consultations outcomes mainly arises from interpersonal factors and are to a lesser extent related to sociocultural norms.

How social concordance between patient and GP influences health care consultations is not clear. However, there are multiple conceivable explanations. First, sharing demographic characteristics can give the persons involved a suggestion of shared identity, ideas and beliefs [5, 12, 27], smoothening for example shared decision making. Secondly, social concordance is associated with a more patient-centered communication style applied by GPs [28–30], which is marked by GPs more actively attempting to understand the patient perspective and reaching shared understanding. Lastly, doctors treating patients who are more like themselves might experience less professional uncertainty because they can better relate to the patient’s problems [5]. These processes could occur simultaneously.

We suggest that future research should focus on more qualitative views on what actually happens in the consultation room. For example, performing qualitative analysis on video recorded consultations could give more insight into consultations room dynamics within each gender dyad. Also, interviewing GPs or patients after a consultation could be an interesting opportunity to unravel how social concordance affects consultation room dynamics.

## Conclusion

We conclude from our study results that age concordance and female/female gender concordance can positively affect PREMs regardless of the gender equality of the countries concerned, although differences were small. This contributes to the idea that the mere act of matching basic demographic characteristics, especially matching a female patient to a female GP, can have a beneficial impact on the experience of healthcare.

### Supplementary Information

**Supplementary Material 1. **

## Data Availability

The raw data used in this study is the property of the international QUALICOPC consortium, and is not available for publication by the authors. The data is available upon reasonable request and can be obtained from W. Schäfer (w.schafer@nivel.nl).

## Abbreviations

PREM: Patient reported experience measure
GEI: Gender equality index
GP: General practitioner
QUALICOPC: Quality and costs of primary care
