# Supplementary material for: Social concordance and patient reported experiences in countries with different gender equality: a multinational survey
Source: BMC Prim Care. 2024 Mar 23;25:97. doi: 10.1186/s12875-024-02339-y (PMC10960425; doi:10.1186/s12875-024-02339-y)
Supplement: Supplementary file 1 — Supplementary Material 1. [file 12875_2024_2339_MOESM1_ESM.docx]

**Appendix A - Constitutes of patient reported outcome measures**

Doctor-patient communication

The doctor was polite (yes/no)

The doctor listened carefully to me (yes/no)

The doctor hardly looked at me when we talked (yes/no)

The doctor asked questions about my health problem (yes/no)

I couldn’t really understand what the doctor was trying to explain (yes/no)

*Reliability: country level 0.65731, GP level 0.86272, patient level 0.73143*

Patient involvement in decision making

The doctor involved me in making decisions about treatment (yes/no)

Comprehensiveness of care

The doctor asked about possible other problems besides the one I just came for (yes/no)

This doctor doesn’t just deal with medical problems but can also help with personal problems
 and worries (yes/no)

*Reliability: country level 0.90488, GP level 0.82203, patient level 0.23563*

Patient satisfaction

The doctor took sufficient time (yes/no)

I would recommend this doctor to a friend or relative (yes/no)

*Reliability: country level 0.78188, GP level 0.82660, patient level 0.62720*
